# Cancer pattern in Bhutan (2014–2022): Findings from population‐based cancer registry—A pathway to monitor cancer control activities

**DOI:** 10.1002/ijc.70308

**Published:** 2026-02-03

**Authors:** Phub Tshering, Ugyen Tshomo, Meera Chetri, Sumitra Suberi, Yangdon Yangdon, Laigden Dzed, Tshewang Lhadon, Sonali Bagal, Sharyu Mhamane, Atul Budukh

**Affiliations:** ^1^ Jigme Dorji Wangchuck National Referral Hospital (JDWNRH) Thimphu Bhutan; ^2^ Department of Public Health Ministry of Health Thimphu Bhutan; ^3^ Centre for Cancer Epidemiology (CCE), Tata Memorial Centre Mumbai Maharashtra India; ^4^ Homi Bhabha National Institute (HBNI), Training School Complex Mumbai India

**Keywords:** Bhutan, cancer control, epidemiology, incidence, population‐based cancer registry

## Abstract

Population‐based cancer registry (PBCR) of Bhutan was established in Jigme Dorji Wangchuk National Referral Hospital (JDWNRH) in 2014 with the support of the Ministry of Health (Bhutan) and IARC Regional Hub, Tata Memorial Centre (TMC), Mumbai, India. This PBCR provides nationwide coverage (0.7 million population). We aim to present the cancer patterns in Bhutan for the years 2014–2022 using PBCR data. Trained registry staff collect cancer patient information by visiting various sources such as hospitals and diagnostic facilities. Data is entered into *CanReg5* software. Data quality and consistency are checked by the IARC Regional Hub‐TMC, Mumbai. The age‐specific rate and age‐adjusted rate were calculated using *CanReg5* software. In the 9‐year period (2014–2022), the PBCR registered 5906 incidence cancer cases, of which 2659 (45%) were males and 3247 (55%) were females. The age‐adjusted incidence rate for males and females was 88.3 and 113.2 per 100,000 population, respectively. Age‐adjusted mortality rates for males and females were 42.7 and 44.6 per 100,000 population, respectively. The leading cancer sites among males are stomach, esophagus, liver, lung, and rectum, and for females, cervix uteri, stomach, breast, lung, and thyroid. Cancer registry will play a pivotal role in boosting and monitoring screening program initiatives in Bhutan. Through effective linkages, it will build a robust database providing a cancer profile of the Bhutanese population which can be employed to devise effective cancer control activities in Bhutan.

## Graphical abstract

What's new?

The establishment in 2014 of the population‐based cancer registry (PBCR) of Bhutan created new opportunities for understanding national cancer trends, which previously were anticipated from data in neighbouring countries. This study presents the first nine‐year summary of cancer patterns in Bhutan, derived from PBCR data. From 2014 to 2022, total recorded incident cancer cases amounted to 5906. One in 10 males and one in nine females were at risk of developing cancer, while one in 19 were at risk of dying. Stomach, oesophagus, and liver were leading cancer sites in males, while cervix, stomach, and breast were leading sites in females.

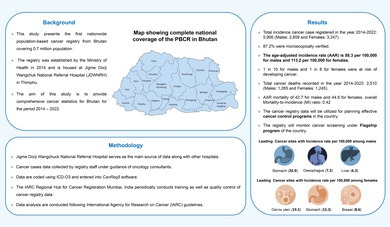

**Abbreviations**
AAR: Age‐adjusted rate
ALD: Alcoholic liver disease
ASR: Age‐specific rate
CCE: Centre for Cancer Epidemiology
GYTS: Global youth tobacco survey
HPV: Human papillomavirus
IARC: International Agency for Research on Cancer
ICD‐O: International Classification of Disease Oncology
JDWNRH: Jigme Dorji Wangchuk National Referral Hospital
LBC: Liquid‐based cytology
LMIC: Lower‐middle‐income country
LSRD: Life Style Related Disease
NE: Northeast
PBCR: Population‐based cancer registry
RICBL: Royal Insurance Corporation of Bhutan Limited
TMC: Tata Memorial Centre
TRC: Tobacco‐related cancer
UK: United Kingdom
USA: United States of America
WHO: World Health Organization

## 1 INTRODUCTION

The overall increase in the cancer burden is attributed to the shift in the epidemiological risk factors. Cancer is the third leading cause of mortality in Bhutan following alcoholic liver disease and cardiovascular disease.^1^ As per GLOBOCAN 2022, the number of incidence cases in 2050 is estimated to be more than double as of 2022. With a rise of 134%, the cases are likely to increase from 638 to 1494. Similarly, mortality is estimated to rise by 144% with an increase from 480 to 1173 cancer deaths annually by the year 2050.^2^ This rising burden of cancer over the years needs to be measured and monitored. Population‐based Cancer Registries (PBCRs) serve as an important tool to gauge and understand the pattern of cancer in a given geographic location. It also plays an important role in monitoring the cancer control activities.^3^

Bhutan is a lower‐middle‐income country (LMIC) in the South‐east Asia region.^4^ The country previously had no cancer registration; however, with the support of the International Agency for Research on Cancer (IARC), Lyon, France; Regional Hub, Tata Memorial Centre (TMC), Mumbai, India, and support from the Ministry of Health of Bhutan, a population‐based cancer registry was established in the year 2014. It is located in Jigme Dorji Wangchuk National Referral Hospital (JDWNRH) in Thimphu, Bhutan. Before the establishment of PBCR Bhutan, data from the neighboring countries was used to anticipate the cancer burden in the country.^5^ The first report of PBCR Bhutan was published in February 2020 for the years 2014–2018 (5 years).^6^

Bhutan has a health care system that is delivered through a three‐tiered health care delivery model with a major thrust in preventive and primary health care services, comprising 27 hospitals, 23 Grade I Basic Health units (BHUs), and 35 sub posts. However, in the absence of comprehensive and current estimates, it is crucial to generate present cancer statistics to support the monitoring of screening activities and to strengthen overall cancer control efforts in the country.^6^

Through this article, we aim to present the cancer patterns in Bhutan for the period of 9 years 2014–2022 using PBCR data, which will serve as a pathway to monitor cancer control activities.

## 2 METHODOLOGY

The IARC Regional Hub‐TMC, Mumbai, India aims to build capacity for cancer registration within the East, South‐East, and South Asia.^7^ One of the countries that the IARC Regional Hub‐TMC, Mumbai supports is Bhutan.

In June 2014, two hospital staff from JDWNRH were trained in the process of establishment, operation and functioning of cancer registries. The capacity building and training of the recruited registry personnel was done at the Centre for Cancer Epidemiology (CCE), TMC, Mumbai, India through a 2‐week training program. At the end of the training, a team of three people consisting of two oncologists and an Assistant Program Officer of the Life Style Related Disease (LSRD) Program visited TMC, Mumbai and worked on the technical aspects of the cancer registry which involved gaining insights on the types of variables to be included or modified in *CanReg5* (A software developed by the IARC, Lyon France for cancer registry operation)^8^ to suit Bhutanese situation. Periodical refresher training and technical support through virtual means are being provided by the IARC Regional Hub‐TMC, Mumbai. To date, the IARC Regional Hub‐TMC, Mumbai has provided 10 training sessions of basic, advanced and refresher training courses. These training sessions include modules on registry methodology, data management, data analysis, survival analysis, report writing, and quality control of the registry.^9^

The PBCR of Bhutan covers every individual in the country with a population of 0.7 million (projected as of 2024). This comprehensive coverage is possible due to Bhutan's small geographic size and its one health system with free health services. By capturing data on every cancer case within the entire population, the registry provides a complete and accurate picture of cancer incidence and trends across the nation. The registry is managed by two nursing staff members and one data entry operator under the supervision and support of a gynecologist and head and neck surgeon.

The data abstracters collect and confirm the information on the vital status of the patient by visiting various sources and register the cancer cases identified either by going through the admission or death records. The major source of this information is from JDWNRH, a tertiary hospital which provides cancer diagnosis, treatment, and other oncology services. The cancer treatment provided to the Bhutanese population is free of cost, even for the citizens who are referred outside of Bhutan.

In addition, other sources include palliative care units, the Bhutan Cancer Society, the Royal Insurance Corporation of Bhutan Limited (RICBL), Regional Referral Hospitals, District Hospitals, and Grade I Basic Health Units (BHUs). Those patients with unknown vital status are annually followed up through telephone. The data abstractors also collect data from all the health centers with admission facilities within the country once a year. This data helps to update the vital status of patients besides recording new cancer cases that have not reached JDWNRH for treatment.

After the information on cancer patients is recorded, data is coded as per the International Classification of Disease Oncology (ICD‐O3) classification.^10^ Before entering the data, it is checked for data quality by the supervisor and the principal investigator. After the data quality is ensured, it is entered into the *CanReg5* software. Duplicates are eliminated through unique ID verification/personal identifiers, telephonic contact as well as through contact with local BHUs staff. Data consistency and quality are checked periodically for duplicates, missing information and other discrepancies by sending it to the IARC Regional Hub‐TMC, Mumbai.

**FIGURE 1:**
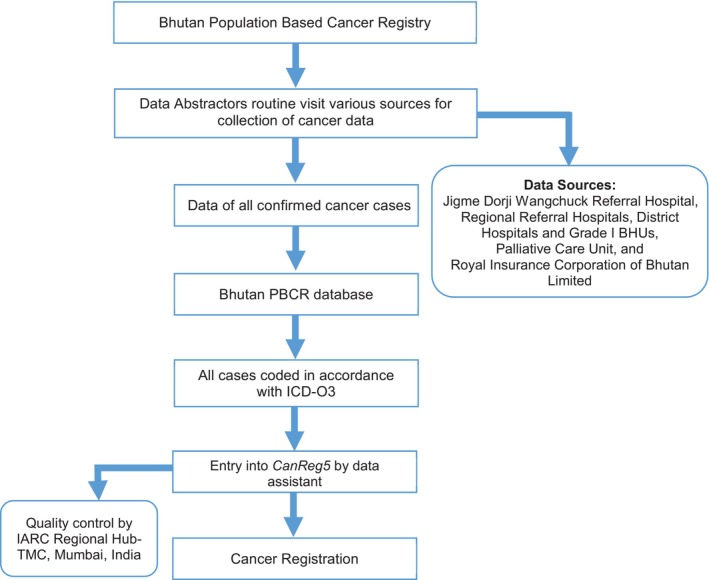
Population‐based cancer registry Bhutan: Methodology.

Although there is no direct face‐to‐face community involvement in the cancer registry, frequent contact was maintained through mobile communication. This was done to confirm diagnosis, and type of treatment undergone and to check the vital status of cancer patients. At the end of each year, particularly for those who were not previously followed up. If patients were found to be alive, they were advised on where to go for follow‐up care. In cases where patients had passed away, the date of death was recorded, and the registry data was updated accordingly. The community cooperation was positive towards the PBCR staff. The detailed methodology of the registry operation is explained elsewhere. Figure 1 illustrates the abovementioned registry methodology. The base population estimates were taken from Census of Bhutan.^11^ The age‐specific rate (ASR), and age‐adjusted rate (AAR) were calculated using *CanReg5* software.^3^, ^8^

The PBCR of Bhutan faces numerous challenges; these include lack of designated space and permanent staff. This leads to several challenges related to data entry and case abstraction. Patients travel abroad for cancer treatment. Hence, these cases are missed by the registry. The challenges are discussed in detail subsequently in the paper. Despite several challenges, the PBCR has presented reliable and good quality data that will be utilized for cancer control activities in Bhutan.

## 3 RESULTS

In the period 2014–2022, a total of 5906 incidence cancer cases were recorded by the PBCR of Bhutan, comprising 2659 (45%) males and 3247 (55%) females. Out of 5906 cancer cases, 87.2% cases were microscopically verified while 9.1% cases had other basis of diagnosis such as radiology and clinical diagnosis, etc. 3.7% of cases were diagnosed by death certificate only (DCO).

The AAR per 100,000 population was 88.3 for males and 113.2 for females. The cumulative risk in males aged 0–74 years was 10.4% (1 in 10 males were at risk of developing cancer) and in females, it was 12.8% (1 in 8 females are at risk of developing cancer).

**FIGURE 2:**
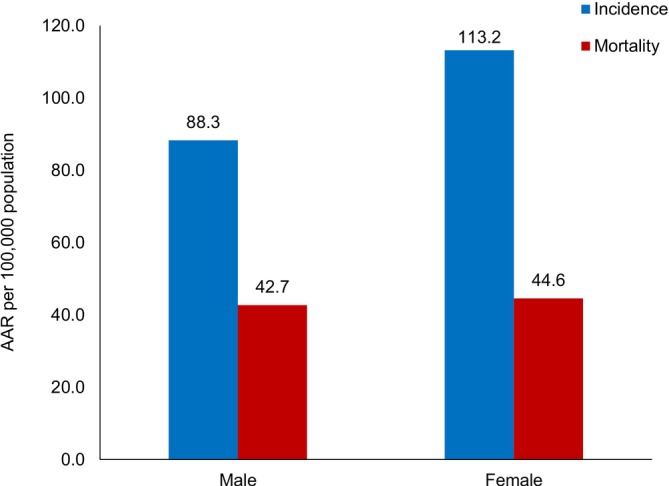
Incidence and mortality of cancer cases in Bhutan: 2014–2022.

A total of 2510 cancer deaths were recorded for the years 2014–2022, of which 1265 (50.4%) were male and 1245 (49.6%) were female cancer deaths. The age‐adjusted mortality rate was 42.7 and 44.6 per 100,000 population for males and females, respectively. The cumulative risk of death due to cancer in males aged 0–74 years was 5.1% (1 in 19 males was at the risk of dying due to cancer) and in females it was 5.4% (1 in 19 women was at risk of dying due to cancer). The cancer incidence and mortality rates in Bhutan for the years 2014–2022 are presented in Figure 2. Males have a mortality–incidence (MI) ratio of 0.47, whereas females have a lower MI ratio of 0.38; the combined MI ratio of both sexes is 0.42.

**FIGURE 3:**
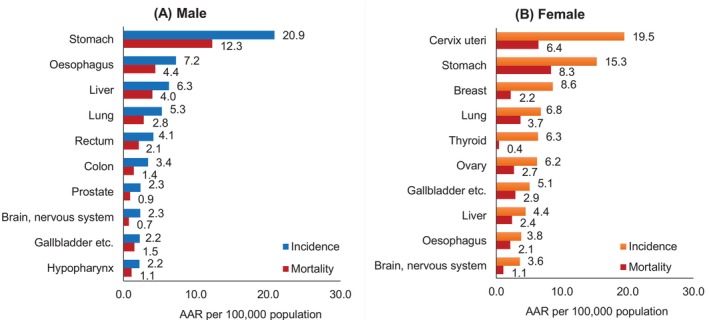
Incidence and mortality rates of leading cancer sites of males and females in Bhutan: 2014–2022.

Table 1 describes the leading cancer sites for incidence and mortality among Bhutanese men and women for the period 2014–2022. The leading cancer sites for males are stomach with an AAR‐20.9 per 100,000 population, esophagus (AAR‐7.2), liver (AAR −6.3), trachea, bronchus and lung (AAR‐5.3), and rectum (AAR −4.1). The leading cancer sites among females were cervix uteri (AAR‐19.5 per 100,000 population), stomach (AAR‐15.3), breast (AAR‐8.6), trachea, bronchus and lung (AAR‐6.8), and thyroid (AAR‐6.3). The leading cancer sites for both males and females are illustrated in Figure 3, respectively. The overall mortality to incidence (MI) ratio for Bhutan PBCR is 0.42 (males: 0.48, females: 0.38).

**TABLE 1:** Average annual age specific, crude (CR), age‐adjusted (AAR), and truncated (35–64 years) (TR) incidence and mortality rate per 100,000 population in males and females: Bhutan (2014–2022).

Incidence in males								Mortality in males							
Cancer Site	Number of cases	%	CR	AAR	TR	Cumulative risk (0–74) (%)	Persons at risk	Cancer Site	Number of cases	%	CR	AAR	TR	Cumulative risk (0–74) (%)	Persons at risk
All cancer sites	2659	100	77.0	88.3	125.4	10.41	1 in 10	All cancer sites	1265	100	36.7	42.7	60.9	5.15	1 in 19
Stomach	613	23.1	17.8	20.9	28.1	2.59	1 in 39	Stomach	357	28.2	10.3	12.3	16.4	1.54	1 in 65
Oesophagus	209	7.9	6.1	7.2	11.5	0.88	1 in 114	Oesophagus	127	10.0	3.7	4.4	7.3	0.55	1 in 182
Liver	185	7.0	5.4	6.3	9.8	0.78	1 in 128	Liver	119	9.4	3.4	4.0	6.1	0.49	1 in 204
Lung	160	6.0	4.6	5.3	7.6	0.63	1 in 159	Lung	85	6.7	2.5	2.8	4.4	0.32	1 in 313
Rectum	123	4.6	3.6	4.1	5.5	0.51	1 in 196	Rectum	63	5.0	1.8	2.1	2.8	0.26	1 in 385

Incidence in females								Mortality in females							
Cancer Site	Number of cases	%	CR	AAR	TR	Cumulative risk (0–74) (%)	Persons at risk	Cancer Site	Number of cases	%	CR	AAR	TR	Cumulative risk (0–74) (%)	Persons at risk
All cancer sites	3247	100.0	103.0	113.2	211.6	12.82	1 in 8	All cancer sites	1245	100.0	39.5	44.6	77.5	5.40	1 in 19
Cervix uteri	569	17.5	18.0	19.5	48.3	2.09	1 in 48	Stomach	235	18.9	7.5	8.3	11.6	1.02	1 in 98
Stomach	426	13.1	13.5	15.3	23.4	1.86	1 in 54	Cervix uteri	181	14.5	5.7	6.4	15.1	0.71	1 in 141
Breast	254	7.8	8.1	8.6	20.8	0.89	1 in 112	Lung	101	8.1	3.2	3.7	5.1	0.51	1 in 196
Lung	187	5.8	5.9	6.8	10.3	0.89	1 in 112	Gallbladder	77	6.2	2.4	2.9	5.3	0.39	1 in 256
Thyroid	212	6.5	6.7	6.3	11.2	0.59	1 in 169	Ovary	75	6.0	2.4	2.7	5.0	0.34	1 in 294

### 3.1 Pediatric cancer rates

The pediatric age group ranges from 0 to 14 years of age. 135 (boys: 69, girls: 66) pediatric cases were registered for the year 2014–2022, accounting for 2.3% of total cases. The incidence in the 0–14 age group is 84.0 per million population for boys and 82.5 for girls. The rates are due to good quality care and treatment provided as well as accurate recording of the pediatric cancer cases in Bhutan.

Table 2 illustrates the comparison of the stomach, liver, lung, breast, and cervical cancer in Bhutan cancer registry with the neighboring cancer registries of Nepal (Kathmandu),^12^ Sri Lanka^13^ and Indian cancer registries of Delhi, Mumbai,^14^ Varanasi,^15^ Muzaffarpur^16^ and Northeast (NE) Indian cancer registries^17^ of Assam (Cachar district, Dibrugarh district, and Kamrup urban), Manipur state, Mizoram state, Sikkim state, Meghalaya, Tripura state, Nagaland, Arunachal Pradesh (West Arunachal, Pasighat) as well as the cancer registries of Thailand, United Kingdom (UK), United States of America (USA), and Curitiba, Brazil.^14^

**TABLE 2:** Comparison of leading cancer sites in Bhutan with neighboring cancer registries of India, Nepal, Sri Lanka, Thailand, United Kingdom and United States of America.

Registry	Males (AAR)			Females (AAR)				
	Stomach	Liver	Lung	Stomach	Liver	Lung	Breast	Cervix Uteri
Bhutan (2014–2022)	20.9	6.3	5.3	15.3	4.4	6.8	8.6	19.5
Kathmandu, Nepal (2019)	8.3	3.2	14.5	4.5	0.5	9.8	17.5	8.3
Sri Lanka (2021)	3.7	4.1	13.7	1.5	1.9	4.0	40.5	9.1
Bangkok, Thailand (2013–2016)	2.5	18.0	20.1	2.2	6.0	9.8	36.3	9.6
Khon Kaen, Thailand (2013–2017)	2.7	39.3	17.1	2.2	12.3	7.5	25.8	8.9
Manipur, India (2012–2016)	4.5	2.3	12.9	2.7	1.2	11.8	10.0	6.7
Imphal West District, India (2012–2016)	3.6	4.5	17.8	3.7	1.0	16.6	16.4	9.4
Mizoram state, India (2012–2016)	39.1	10.0	32.1	18.8	5.9	27.6	21.6	23.2
Aizwal district, India (2012–2016)	44.2	12.2	38.8	21.7	4.8	37.9	30.7	27.4
Sikkim state, India (2012–2016)	15.7	6.4	6.5	7.9	4.9	7.1	10.6	9.9
Tripura state, India (2012–2016)	5.0	1.9	14.5	2.2	1.3	3.4	7.9	9.5
West Arunachal, India (2012–2016)	24.9	21.5	7.0	15.8	8.0	5.0	10.2	10.0
Papumpare district, India (2012–2016)	40.3	35.2	20.1	27.1	14.4	12.8	29.6	27.7
Meghalaya, India (2012–2016)	12.2	3.6	12.4	6.9	1.4	4.3	7.0	8.8
East Khasi Hills district, India (2012–2016)	13.6	4.5	14.1	8.0	1.9	5.3	9.0	9.4
Nagaland, India (2012–2016)	17.9	4.1	8.4	11.8	2.0	4.3	9.2	13.3
Pasighat, India (2012–2016)	23.9	7.1	9.7	12.5	5.2	–	17.8	20.3
Cachar district, India (2012–2016)	5.6	1.7	11.9	3.4	1.1	3.9	14.0	15.3
Dibrugarh district, India (2012–2016)	7.0	3.7	5.1	4.1	1.9	2.0	14.7	4.8
Kamrup Urban, India (2012–2016)	13.4	6.8	18.1	7.9	3.3	6.7	27.1	14.2
Delhi, India (2013–2017)	3.8	4.7	17.0	2.5	2.3	5.0	37.1	13.0
Mumbai, India (2013–2017)	4.6	6.8	10.5	2.8	3.3	5.8	34.2	7.9
Varanasi, India (2018–2019)	2.6	3.8	3.8	1.4	2.4	2.2	13.1	7.2
Muzaffarpur, India (2018)	2.0	1.6	1.2	0.9	1.1	1.3	12.5	7.4
England, United Kingdom (2013–2017)	6.1	6.1	35.2	2.7	2.6	27.5	92.4	7.7
United States of America (2013–2017)	5.6	9.4	41.1	3.0	3.1	32.3	91.0	6.0
Curitiba, Brazil (2013–2017)	13.1	6.2	16.2	5.6	2.6	10.9	52.6	11.1

*Note*: The colour gradient signifies the magnitude of incidence, deeper colours represent higher incidence and vice versa.

For males among the above‐mentioned cancer registries, the burden for given cancers was high in northeast (NE) cancer registries of India. After NE Indian cancer registries, Bhutan had the highest AAR for stomach cancer (20.9 per 100,000 population) followed by Curitiba, Brazil (13.1 per 100,000 population) while Muzaffarpur had the lowest rate of stomach cancer (2 per 100,000 population). Similar observations were seen for females.

For females among the above‐mentioned cancer registries, the rate for stomach cancer in Bhutan (15.3 per 100,000 population) was comparable to NE Indian states, with the lowest in Muzaffarpur (0.9 per 100,000 population). The highest rates for breast cancer were recorded for the UK (92.4 per 100,000 population), and the lowest in Meghalaya, India (7 per 100,000 population). However, Bhutan reported the highest rate of cervical cancer among these registries (19.5 per 100,000 population), while the lowest was in the USA (6 per 100,000 population).

The anatomical sites are included to calculate the tobacco‐related cancer burden based on the guidelines of the IARC, Lyon, France. These sites include lip (C00), tongue (C01‐C02), mouth (C03‐C06), pharynx (C10, C12‐C14), esophagus (C15), larynx (C32), lung (C33‐C34), and urinary bladder (C67) as these are associated with the use of tobacco.^18^

The proportion of tobacco‐related cancer (TRC) is also compared with neighboring cancer registries of India,^14^, ^15^, ^16^, ^17^ Nepal,^12^ Sri Lanka,^13^ United Kingdom, and United States of America.^14^ The details are illustrated in Table 3.

**TABLE 3:** Comparison of tobacco‐related cancer in Bhutan with neighboring cancer registries of India, Nepal, Sri Lanka, Thailand, United Kingdom and United States.

Registry	Males		Females	
	%	AAR per 100,000	%	AAR per 100,000
Bhutan (2014–2022)	25.0	22.5	13.6	16.2
Kathmandu, Nepal (2019)	35.3	23.2	17.3	12.7
Sri Lanka (2021)	43.4	66.5	12.6	18.2
Khon Kaen, Thailand (2013–2017)	20.6	27.1	10.3	11.0
Bangkok, Thailand (2013–2016)	27.5	35.0	10.9	13.5
Manipur, India (2012–2016)	36.8	24.7	19.5	15.8
Imphal West District, India (2012–2016)	37.3	36.8	19.1	22.2
Mizoram state, India (2012–2016)	43.3	89.3	22.1	42.3
Aizwal district, India (2012–2016)	47.2	127.1	24.4	56.9
Sikkim state, India (2012–2016)	32.8	29.5	18.2	19.2
Tripura state, India (2012–2016)	52.1	43.2	21.1	13.0
West Arunachal, India (2012–2016)	24.5	26.6	11.1	13.7
Papumpare district, India (2012–2016)	30.5	67.7	14.4	43.6
Meghalaya, India (2012–2016)	66.9	119.7	43.1	44.6
East Khasi Hills district, India (2012–2016)	70.4	161.3	46.5	58.1
Nagaland, India (2012–2016)	39.3	51.1	11.5	12.5
Pasighat, India (2012–2016)	29.0	36.1	10.9	14.5
Cachar district, India (2012–2016)	54.0	71.2	23.4	26.9
Dibrugarh district, India (2012–2016)	51.8	48.9	21.8	18.2
Kamrup Urban, India (2012–2016)	51.6	110.2	23.5	43.2
Delhi, India (2012–2016)	41.2	62.1	12.4	18.5
Mumbai, India (2012–2016)	38.7	41.8	15.6	18.2
Varanasi, India (2018–2019)	50.6	36.8	13.1	7.8
Muzaffarpur, India (2018)	36.6	15.0	8.8	4.2
England, United Kingdom (2013–2017)	18.4	77.4	13.1	41.6
United States of America (2013–2017)	26.0	77.9	17.0	44.3
Curitiba, Brazil (2013–2017)	18.7	47.0	8.6	17.5

As compared to the neighboring cancer registries and registries from the West, the proportion of TRC is higher in the Indian and Bhutan cancer registries as compared to the Thailand, UK, USA, and Curitiba, Brazil. However, rates of TRC were higher in cancer registries of developed countries as opposed to those in developing countries. These findings were similar among both sexes.

## 4 DISCUSSION

The PBCR of Bhutan faces numerous challenges due to its limited health infrastructure and human resources. These include lack of designated space and staff for cancer registry for optimal functioning; lack of regularized/full‐time staff leads to hindrances in data entry and case abstraction, which then leads to missing information due to incomplete documentation. The medical records are manually maintained, furthering the scope of missing information. The geography of Bhutan makes it difficult for cancer patients to travel across for cancer treatment. Although all the cancer treatment expenses in and out of the country are borne by the government, some cancer patients travel to India or other countries for treatment at their own expense, imposing additional financial burden on the cancer patients. These cases have a chance to be missed from our registry database. Additionally, there are no designated days for cancer follow‐up in Bhutan. Due to that, updating vital status is difficult. Despite these challenges, the PBCR has managed to provide reliable and good‐quality data on cancer in Bhutan. Data is checked periodically by the supervisor and sent to the IARC Regional Hub‐TMC, Mumbai for standard quality check. The data quality indicators as per the IARC standards for good quality cancer registry data are described elsewhere.^19^

The risk of getting cancer in Bhutan is one in 10 for males and one in eight for females. This is similar to that noted in Kathmandu, Nepal, that is, one in 10 for both males and females, respectively. However, the risk of developing cancer is higher in Sri Lanka (males: 1 in 5; females: 1 in 6) and most of the northeastern states of India such as Kamrup Urban (males: 1 in 4; females: 1 in 6), Mizoram state (males: 1 in 5; females: 1 in 5), and Meghalaya (males: 1 in 5; females: 1 in 9) to name a few. The risk is comparable to Sikkim, Tripura, and Manipur states of India. The Mortality–incidence (MI) ratios of Bhutan are higher in males than females. Meaning in males roughly 47.6% of incident cancer cases result in death, whereas females have a lower MI ratio indicating about 38.3% of cases lead to mortality. When both sexes are combined, the overall MI ratio reflects that over 42.5% of all cancer cases result in death, suggesting that cancers in men tend to have poorer outcomes compared to women.

The cancer incidence profile of Bhutan reveals distinct variations compared with neighboring South Asian countries and selected global populations. Among males, the AAR for stomach cancer in Bhutan (20.9 per 100,000) is substantially higher than in Nepal (8.3), Sri Lanka (3.7), Thailand (2.5–2.7), the United States (5.6), the United Kingdom (6.1), and Brazil (13.1), though lower than in several northeastern regions of India such as Mizoram (39.1) and Aizawl (44.2). The liver cancer incidence in Bhutanese men (6.3) is comparable to that of Sri Lanka (4.1) and higher than in Nepal (3.2), but markedly lower than in Khon Kaen, Thailand (39.3). For lung cancer, Bhutanese males (5.3) exhibit a much lower incidence than Nepal (14.5), Sri Lanka (13.7), northeastern India (up to 38.8 in Aizawl), Thailand (17.1–20.1), the United States (41.1), and the United Kingdom (35.2).

Among females, Bhutan reports higher rates of stomach (15.3) and cervix uteri (19.5) cancers compared with Nepal (4.5 and 8.3), Sri Lanka (1.5 and 9.1), Bangkok (Thailand) (2.2 and 9.6), the United States (3.0 and 6.0), the United Kingdom (2.7 and 7.7), and Brazil (5.6 and 11.1), respectively. In contrast, breast cancer incidence in Bhutanese women (8.6) remains significantly lower than in Sri Lanka (40.5), Thailand (25.8–36.3), India's urban areas such as Delhi (37.1) and Mumbai (34.2), as well as the United States (91.0), the United Kingdom (92.4), and Brazil (52.6). Overall, Bhutan's cancer pattern aligns more closely with other South Asian countries, characterized by relatively higher rates of infection‐related cancers such as stomach and cervical cancers, and comparatively lower rates of breast cancer typically seen in high‐income countries.

Our analysis of the population‐based cancer registry Bhutan reveals a distinct pattern of cancer incidence, with the stomach, esophagus, and liver as the leading cancer sites among males while cervix uteri, stomach, and breast as the leading sites among females. Notably, the data indicates a burden of cancers associated with lifestyle changes and urbanization. Providing health education and awareness about the associated risk factors of common cancer in Bhutan would be of prime importance to curtail cancer in this region.

### 4.1 Gastric cancer

Among both sexes, gastric cancer is the predominant cancer which is closely attributed to Helicobacter Pylori (*H. pylori*) infection. The prevalence of *H. pylori* infection is established to be higher in developing countries (82%) than in developed countries (40%).^20^, ^21^, ^22^ This is due to poor hygiene and sanitation practices. Barely 63% of Bhutanese have access to basic sanitation services.^23^, ^24^ However, in recent years there has been improvement in *H. pylori* control due to the evolution of various antibiotic and treatment modalities for *H. pylori*. In addition to the treatment, screening and eradication of *H. pylori* through a screening program in Bhutan is recommended.^25^ From 2020 to 2023, a comprehensive nationwide cancer screening program for breast, cervical and gastric cancers was implemented. A total of 91.2% of the target population (18–75 years) were covered under this program. Of the 370,225 screened for *H. Pylori*, 32.4% tested positive; among these, 53,182 underwent upper gastrointestinal endoscopy and biopsy from which 255 (0.07%) gastric cancers were diagnosed.^24^ These cancer cases can be further followed up by the PBCR via adequate linkages.

The association between *H. pylori* infection and gastric cancer is well established,^26^, ^27^, ^28^ but the high prevalence of *H. pylori* infection is not always associated with high incidence of gastric cancer. For example, despite the high *H. pylori* infection rate in India, the incidence of gastric cancer is low, a phenomenon that has been termed the “Asian enigma”.^29^

### 4.2 Cervical cancer

The high rates of cervical cancer are centrally attributed to the occurrence of human papillomavirus (HPV) infections. As this viral transmission is through the sexual route, women with multiple sexual partners, multiple pregnancies, or early initiation of sexual activities are the attributable risk factors while other risk factors include other genital infections and smoking.^30^, ^31^ This can also include poor sanitation and hygiene practices.

Cervical cancer rate is high in Bhutan as compared to the neighboring countries which might be due to the screening program.^32^ Bhutan is committed to achieving the 90–70–90 goals by 2030, which are 90% HPV vaccination coverage, 70% twice‐lifetime cervical screening, and 90% treatment of preinvasive and invasive cervical lesions.^33^ To tackle this issue, Bhutan commenced a nationwide school‐based program of vaccination against HPV since 2010 and was the first LMIC to roll out such a program.^34^ The emphasis was laid on school‐going girls as more than 30% of the burden of cervical cancer was borne by girls younger than 14 years.^35^

From 2020 to 2023, Bhutan launched a cervical cancer elimination program in line with the World Health Organization's worldwide strategy for expedited cervical cancer elimination. The Health Flagship Program aimed to screen women aged 30–65 years with HPV testing using liquid‐based cytology (LBC) as triaging for screen‐positive women. Bhutan adopted a gender‐neutral vaccination program by initiating vaccination of adolescent boys beginning in September 2020 through its well‐established network of primary healthcare centres.^36^

In addition to this, the Flagship Program also carried out a comprehensive nationwide screening for breast and stomach cancers along with cervical cancer as Bhutan's cancer control strategy.^24^

### 4.3 Liver cancer

Alcohol consumption is an inseparable part of the Bhutanese culture. Various forms of alcohol are prepared and often served as hospitality in Bhutan. Alcoholic liver disease (ADL) is among the top three leading causes of mortality in Bhutan.^25^, ^37^ ALD is associated with a more than two‐fold increased risk of liver cancer, making it a possible risk factor for the burden of liver cancer in Bhutan. Alcohol is classed as a Group I carcinogen; efforts have been made to limit alcohol consumption by the Royal Government of Bhutan to control ALD. These include banning the production of household alcohol, raising taxation on factory‐produced alcohol, increasing the legal alcohol consumption age from 18 to 21, and a dry day in the week on every Tuesday. The government has an ongoing advocacy activity on the ill effects of alcohol among rural and urban areas. These measures are formulated to regulate and prevent the alcohol‐related problem in Bhutan.^37^

### 4.4 Lung cancer

The highest burden of lung cancer is recorded to be in South‐east Asia.^38^ The substantial proportion of those diagnosed with lung cancers in South‐east Asia are never‐smokers.^39^ Therefore, in such cases the risk factors include exposure to indoor and outdoor air pollution (particulate matter), second‐hand smoke, and occupational exposure to asbestos in the mining, cement, and other construction‐related industries, as well as to chromium, cadmium, arsenic, and coal products. In South‐east Asia, second‐hand smoke from burning biomass fuels at home is prevalent in rural and hilly areas. The increased risk of lung cancer among never‐smokers has also been linked to variables such as hormonal state, genetic vulnerability, and pre‐existing lung illness or past history of tuberculosis.^39^

### 4.5 Tobacco related cancers

Bhutan has a moderate tobacco‐related cancer burden among men when compared to its neighboring nations; it is lower than Thailand and Brazil, and extremely high rates were reported in northeastern India (such as Aizawl, Meghalaya, and East Khasi Hills), the USA, and the UK. Bhutanese women have AARs that are comparable with Nepal, Sri Lanka, and Thailand. However, they are marginally lower than those of certain nations such as India (such as Aizawl, Meghalaya, and East Khasi Hills), the USA, and the UK.

In addition, tobacco consumption is a crucial risk factor. Smokeless form of tobacco is mostly preferred in the South‐east Asia Region as opposed to the smoked form used in high‐income countries. The South‐east Asian countries are substantially affected by the tobacco‐related morbidity and mortality. India, being the leading consumer and producer, has its concentration of tobacco‐related cancers in the northeast. The neighboring border of Bhutan with the northeast state of India reflects similar tobacco consumption habits. For example, fermented betel nut is chewed in Bhutan as well as in NE India under different names.^40^

Chewing areca nut is culturally ingrained among the Bhutanese population. Fermented areca nut (Doma) is widely consumed in Bhutan, with about 45% of Bhutanese men consuming it. The process of preparing Doma involves fermentation of areca nut using raw materials: areca nut, jute bag, and cow dung. Fermentation increases the carcinogenic properties of areca nut, which is tightly linked to the cancer of oral cavity and oropharynx.^40^ These practices are common and are seen to begin from a very early age. As per Global youth tobacco survey (GYTS) 2019 report, one out of five students in Bhutan are current tobacco users, with the proportion higher in boys (31.2%) than girls (13.5%).^41^ Bhutan is the only country in the world to levy a comprehensive ban on cultivation, manufacturing, and distribution of tobacco, meaning those who wish to consume tobacco have to import it in limited quantity.^42^ Though government taxes tobacco imports in Bhutan heavily, the proportion of youth consuming tobacco is a matter of concern. Apart from health hazard, tobacco imposes a deep economic loss. In 2019, tobacco use in Bhutan caused 1.2 billion Bhutanese Ngultrum (BTN) in economic loss, which was 0.7% of Bhutan GDP.^43^

### 4.6 Bhutan cancer control strategy

In line with the cancer control strategy of the Royal Government of Bhutan, the Ministry of Health implemented the Health Flagship Programme. This is one of the nine flagship initiatives for its 12th Five‐Year Plan (2018–2023). This initiative was a countrywide cancer screening program that ran from 2020 to 2023 and cost Nu 1109.572 million (US$ 13.095).^44^ Health finance in Bhutan is often linked to the annual budget allocation from the projected Five‐Year Plan. A specific budget was set aside from the government's flagship budget to undertake the Health Flagship Programme.^45^ This initiative focused on screening for three cancers: gastric, cervical, and breast, with the ultimate goal of lowering incidence and death. This was a population‐level screening effort, followed by linking test‐positive persons to appropriate care.^25^ This ambitious initiative had excellent screening coverage despite being operational at the population level and has led to improved access to cancer screening. The success of which was attributed to Bhutan's political commitment, community engagement and comprehensive advocacy program.^46^ The National Cancer Control Programme must devise novel strategies for keeping patients in the screening cascade and providing enough follow‐up services. Bhutan has reached 90–70–90 World Health Organization objectives for cervical cancer eradication as of 2023,^33^ however maintaining this level of accomplishment would need further coordinated efforts.^47^ Policy interventions for south‐east Asian countries like Bhutan are often misguided and rely heavily on past interventions rolled out in high income countries without accounting for the differences in the risk factor exposures in these diasporas.^48^

### 4.7 Cancer registry for cancer control in Bhutan

The gold standard for providing data on cancer incidence in a specific group is PBCRs; it can be used to determine potential etiology of cancer in the community, evaluate the effectiveness of cancer management initiatives, and build the foundation for epidemiological studies.

PBCR data also enable the identification of tobacco‐related cancers, supporting the development of targeted strategies to reduce their impact. Similarly, PBCRs can monitor HPV‐related cancers and assess the effectiveness of prevention efforts such as vaccination programs and screening practices. For instance, in Bhutan, the flagship program focusing on screening for gastric, cervical, and breast cancers can be effectively monitored through data collected by the registry. The existing efforts to curtail cancer control efforts can be boosted by using a population‐based cancer registry and its linkages to the existing as well as the past cancer control program. The individuals detected as screen positives during the screening programs can be further followed up and monitored by the Bhutan PBCR.

This will help in building a proper network of cancer care pathway in the nation and will help in achieving a robust database providing a detailed cancer profile of the Bhutanese population which will be further used in formulating effective cancer control strategies in the future.

Bhutan serves as an ideal example of a low‐resource setting where the entire population is covered under its PBCR. To ensure the sustainability of PBCRs in LMICs, the Ministry of Health should take proactive initiatives and allocate adequate funding. The registry should preferably be located within a cancer center. Consultants should be actively involved, with technical support provided by the IARC regional hub. The success of PBCRs also depends on active community involvement and collaboration among clinicians, oncologists, and administrative staff to ensure accurate and timely access to data.

In addition to this, cancer registries will serve as an adjunct tool to assist with the follow‐up of the cases identified during the health flagship program. The Bhutan cancer registry, along with the technical support from the IARC Regional Hub‐TMC, Mumbai, will devise effective strategies to link the existing cancer control activities that will aid in follow‐up and monitoring the cancer control activities in Bhutan.

## 5 CONCLUSION

In conclusion, this study sheds light on the existing patterns of cancer in Bhutan, highlighting both the incidence and mortality of specific cancer types predominant in the population. The findings emphasize the need for strengthening the already established national cancer registry to facilitate ongoing cancer surveillance, control strategies, and research.

The results emphasize the importance of accurate cancer data for directing evidence‐based policy decisions and public health initiatives. However, challenges in data collection and provider coordination must be addressed to ensure the smooth functioning of the registry.

It is important to note that maintaining and sustaining PBCR operations requires ownership from consultants, consistent funding from the ministry of health, regular and refresher training, quality control by IARC hub or expert groups.

In addition to this, by addressing lifestyle factors, education in regards to commonly occurring cancers, preventive measures and improving access to healthcare, Bhutan can enhance its response to cancer, ultimately improving outcomes for patients. Continued collaboration between healthcare providers, policymakers, and researchers will be crucial in combating this growing health challenge and ensuring a healthier future for all Bhutanese.

Additionally, the insights gained underscore the importance of targeted prevention and early detection programs tailored to the Bhutanese population. The study hints at data linkage between the PBCR database and the existing comprehensive cancer screening program to develop a robust database, essential follow‐up and monitoring that will prove to be an important step to level up cancer control efforts. For bringing the cancer control initiatives of the government to fruition, the cancer registry will serve as an excellent pathway to monitor cancer control activities in Bhutan.

## AUTHOR CONTRIBUTIONS

**Phub Tshering:** Conceptualization; data curation; writing – original draft; validation. **Ugyen Tshomo:** Conceptualization; validation; writing – original draft; data curation. **Meera Chetri:** Data curation; validation; writing – review and editing. **Sumitra Suberi:** Validation; data curation; writing – review and editing. **Yangdon Yangdon:** Validation; data curation; writing – review and editing. **Laigden Dzed:** Validation; data curation; writing – review and editing. **Tshewang Lhadon:** Validation; data curation; writing – review and editing. **Sonali Bagal:** Formal analysis; validation; writing – review and editing. **Sharyu Mhamane:** Validation; writing – review and editing; formal analysis. **Atul Budukh:** Conceptualization; validation; writing – review and editing.

## FUNDING INFORMATION

Jigme Dorji Wangchuk National Referral Hospital (JDWNRH), Ministry of Health, Bhutan.

## CONFLICT OF INTEREST STATEMENT

The authors declare no conflict of interest.

## ETHICS STATEMENT

This study has been approved by REBH (Research and Ethical board for health ministry) of Bhutan (Ethical number 2025.89.NNW). The data used is collected by the registry, as a part of their routine surveillance program; therefore, a waiver of consent was granted.

## Data Availability

Derived data supporting the findings of the study and further information are available from the corresponding author Dr. Phub Tshering [ptshering@jdwnrh.gov.bt] upon reasonable request.

## References

[1] Ministry of Health . Annual Health Bulletin. Policy and Planning Division (PPD), Ministry of Health (MoH), Royal Government of Bhuta. 2022 https://moh.gov.bt/wp-content/uploads/2025/01/Annual-Health-Bulleti-2022_Link-3.pdf

[2] Ferlay J , Ervik M , Lam F , et al. Global Cancer Observatory. International Agency for Research on Cancer, Lyon, France. 2024 https://gco.iarc.fr/

[3] Jensen OM , Parkin DM , MacLennan R , Muir CS , Skeet RG . Cancer registration: principles and methods (IARC Scientific Publications No. 95). International Agency for Research on Cancer, World Health Organization, Lyon, France. 1991.

[4] The World Bank in Bhutan . 2025. https://www.worldbank.org/en/country/bhutan/overview

[5] Cancer Incidence and Mortality in Bhutan: 2014–2018 . Report of the Population Based Cancer Registry Bhutan Jigme Dorji Wangchuck National Referral Hospital (JDWNRH), Thimphu, Bhutan. 2020.

[6] Tshomo U , Tshering P , Bagal S , Budukh A . Cancer Incidence and Mortality in Bhutan: 2019–2022. National Cancer Control Program, Department of Public Health, Ministry of Health, Thimphu, Bhutan. 2024 https://moh.gov.bt/wp-content/uploads/2025/07/A4_Final_PBCR-19-22-report.pdf

[7] The Global Initiative for Cancer Registry Development . The Global Initiative for Cancer Registry Development. 2024 https://gicr.iarc.fr/about-the-gicr/ 10.21149/15303 39977063

[8] International Agency for Research on Cancer . CanReg5 Open Source Software (Lyon, France: International Agency for Research on Cancer). 2008 http://www.iacr.com.fr/index.php?option=com_content&view=article&id=9:canreg5&catid=68&Itemid=445

[9] Budukh A , Dikshit R . IARC Regional Hub for Cancer Registration, Mumbai – Journey over the Decade 2012–2022, Tata Memorial Centre (TMC), Mumbai, India. 2023.

[10] World Health Organization (WHO) . International Classification of Diseases for Oncology 3rd edn (Geneva: WHO). 2000 https://www.who.int/standards/classifications/other-classifications/international-classification-of-diseases-for-oncology

[11] Census Report – National Statistics Bureau, Bhutan. 2025 https://www.nsb.gov.bt/publications/census-report/

[12] Dhimal M , Dahal U , Khadka K , et al. Cancer incidence and mortality in selected districts of Nepal in 2019. Nepal Health Research Council. 2022 https://nhrc.gov.np/wp-content/uploads/2022/08/Population-Based-Cancer-Registry-Report-2019-1.pdf

[13] National Cancer Incidence and Mortality Data Sri Lanka, 2021 . Colombo: National Cancer Control Programme, Ministry of Health. 2023 https://www.nccp.health.gov.lk/storage/post/pdfs/CANCER%20INCIDENCE%20&%20MORTALITY%20DATE%20SRI%20LANKA%202021.pdf

[14] Bray F , Colombet M , Aitken JF , et al. In: Ferlay J , ed. Cancer Incidence in Five Continents Vol. XII. International Agency for Research on Cancer; 2023. https://ci5.iarc.who.int

[15] Budukh A , Khanna D , Bagal S , et al. Cancer Incidence and Mortality in Varanasi District, Uttar Pradesh, India: 2018–2019. Tata Memorial Centre; 2022.

[16] Budukh A , Bagal S , Pandey N , et al. Cancer Incidence and Mortality in Muzaffarpur, Bihar State, India: 2018. Tata Memorial Centre (TMC), Mumbai, India. 2023 https://tmcepi.gov.in/assets/Documents/MRR_Reports/Muzaffarpur%20PBCR_2018.pdf

[17] Director, Indian Council of Medical Research (ICMR)‐National Centre for Disease Informatics and Research (NCDIR), Bengaluru, India . Report On Sites of Cancer Associated with Tobacco Use in India. 2021 https://ncdirindia.org/All_Reports/TRC_Report/resources/TRC_eBook.pdf

[18] IARC . IARC Monographs—Supplement 7, Overall Evaluations of Carcinogenicity: an Updating of IARC Monographs Volume 1–42: IARC monographs on the evaluation of the carcinogenic risks to humans. IARC; 1987:357‐361. 3482203

[19] Bray F , Znaor A , Cueva P , et al. Planning and Developing Population‐Based Cancer Registration in Low‐ or Middle‐Income Settings. Lyon (FR): International Agency for Research on Cancer; (IARC Technical Report, No. 43.) Chapter 5., quality control at the population‐based cancer registry. 2014 https://www.ncbi.nlm.nih.gov/books/NBK566960/ 33502836

[20] Fock KM , Ang TL . Epidemiology of helicobacter pylori infection and gastric cancer in Asia. J Gastroenterol Hepatol. 2010;25(3):479‐486. doi:10.1111/j.1440-1746.2009.06188.x 20370726

[21] Shiota S , Mahachai V , Vilaichone RK , et al. Seroprevalence of helicobacter pylori infection and gastric mucosal atrophy in Bhutan, a country with a high prevalence of gastric cancer. J Med Microbiol. 2013;62(Pt 10):1571‐1578. doi:10.1099/jmm.0.060905-0 23831768 PMC3799224

[22] Vilaichone RK , Aumpan N , Ratanachu‐Ek T , et al. Population‐based study of helicobacter pylori infection and antibiotic resistance in Bhutan. Int J Infect Dis. 2020;97:102‐107. doi:10.1016/j.ijid.2020.05.077 32474200

[23] WASH: Water, Sanitation and Hygiene . UNICEF for every child, Bhutan. 2025 https://www.unicef.org/bhutan/wash-water-sanitation-and-hygiene/maternal-newborn-and-child-health/wash-water-sanitation-and-0-0-1

[24] Chophel T , Tshering S , Dorji N , Tshomo U . Stomach cancer screening Services of Bhutan. Indian J Surg. 2022;23:1‐6. doi:10.1007/s12262-022-03519-9 PMC9307435 35912395

[25] Dorji T , Wangmo S , Dargay S , et al. Population‐level cancer screening and cancer care in Bhutan, 2020–2023: a review. Lancet Reg Health Southeast Asia. 2024;24:100370. doi:10.1016/j.lansea.2024.100370 38444883 PMC10910341

[26] Trang TT , Shiota S , Matsuda M , et al. The prevalence of helicobacter pylori virulence factors in Bhutan, Vietnam, and Myanmar is related to gastric cancer incidence. Biomed Res Int. 2015;2015:830813. doi:10.1155/2015/830813 26090448 PMC4450262

[27] Uemura N , Okamoto S , Yamamoto S , et al. *Helicobacter pylori* infection and the development of gastric cancer. N Engl J Med. 2001;345(11):784‐789. doi:10.1056/NEJMoa001999 11556297

[28] Parsonnet J , Friedman GD , Vandersteen DP , et al. Helicobacter pylori infection and the risk of gastric carcinoma. N Engl J Med. 1991;325(16):1127‐1131. doi:10.1056/NEJM199110173251603 1891020

[29] Miwa H , Go MF , Sato N . *H. pylori* and gastric cancer: the Asian enigma. Am J Gastroenterol. 2002;97(5):1106‐1112. doi:10.1111/j.1572-0241.2002.05663.x 12014714

[30] Louie KS , de Sanjose S , Diaz M , et al. Early age at first sexual intercourse and early pregnancy are risk factors for cervical cancer in developing countries. Br J Cancer. 2009;100(7):1191‐1197. doi:10.1038/sj.bjc.6604974 19277042 PMC2670004

[31] Cervical Cancer Causes, Risk Factors, and Prevention: National Cancer Institute. August 2, 2024. 2024 https://www.cancer.gov/types/cervical/causes-risk-prevention

[32] Parikh PM , Mullapally SK , Hingmire S , et al. Cervical cancer in SAARC countries. South Asian J Cancer. 2023;12(1):1‐8. doi:10.1055/s-0043-1764227 36851937 PMC9966176

[33] Global strategy to accelerate the elimination of cervical cancer as a public health problem. Global strategy (17 November 2020). 2025 https://www.who.int/publications/i/item/9789240014107

[34] Baussano I , Tshomo U , Clifford GM , Tenet V , Tshokey T , Franceschi S . Cervical cancer screening program in Thimphu, Bhutan: population coverage and characteristics associated with screening attendance. BMC Womens Health. 2014;14:147. doi:10.1186/s12905-014-0147-0 25433538 PMC4258285

[35] Hingmire S , Tshomo U , Dendrup T , Patel A , Parikh P . Cervical cancer HPV vaccination and Bhutan. South Asian J Cancer. 2023;12(1):41‐43. doi:10.1055/s-0043-1764220 36860586 PMC9970746

[36] Dorji T , Tshomo U , Gyamtsho S , Tamang ST , Wangmo S , Pongpirul K . Gender‐neutral HPV elimination, cervical cancer screening, and treatment: experience from Bhutan. Int J Gynaecol Obstet. 2022;156(3):425‐429. doi:10.1002/ijgo.13728 33930178

[37] Wangchuk P . Burden of alcoholic liver disease: Bhutan scenario. Euroasian J Hepatogastroenterol. 2018;8(1):81‐82. doi:10.5005/jp-journals-10018-1267 29963471 PMC6024037

[38] Pakzad R , Mohammadian‐Hafshejani A , Ghoncheh M , Pakzad I , Salehiniya H . The incidence and mortality of lung cancer and their relationship to development in Asia. Transl Lung Cancer Res. 2015;4(6):763‐774. doi:10.3978/j.issn.2218-6751.2015.12.01 26798586 PMC4700233

[39] Noronha V , Budukh A , Chaturvedi P , et al. Uniqueness of lung cancer in Southeast Asia. Lancet Reg Health Southeast Asia. 2024;27:100430. doi:10.1016/j.lansea.2024.100430 39157507 PMC11328770

[40] Patel A , Patel M , Tshering P , Koyyala VPB , Ghadyalpatil N . Chewing Doma (fermented betel nut): culture versus cancer? South Asian J Cancer. 2023;13(1):1‐3. doi:10.1055/s-0043-1764216 38721101 PMC11076091

[41] Global Youth Tobacco Survey (GYTS), Bhutan 2019. 2025 https://www.who.int/publications/i/item/9789290227724

[42] Gurung MS , Pelzom D , Dorji T , et al. Current tobacco use and its associated factors among adults in a country with comprehensive ban on tobacco: findings from the nationally representative STEPS survey, Bhutan, 2014. Popul Health Metr. 2016;14:28. doi:10.1186/s12963-016-0098-9 27507928 PMC4977656

[43] United Nations Development Programme Bhutan . 2025 https://www.undp.org/

[44] BLUEPRINT‐HEALTH‐FLAGSHIP‐PROJECT.pdf. 2025 https://moh.gov.bt/wp‐content/uploads/2025/02/BLUEPRINT‐HEALTH‐FLAGSHIP‐PROJECT.pdf

[45] Twelfth Five Year Plan 2018‐2023. Volume II: Central Plans. Gross National Happiness Commission, Royal Government of Bhutan. 2018 https://planipolis.iiep.unesco.org/sites/default/files/ressources/bhutan_12fyp‐volume‐ii‐central‐plans.pdf

[46] Pempa , Dorji T , Tashi U , Choden J , Dema C , Dorji T . Implementation of a nationwide population‐level cancer screening in Bhutan: a programmatic experience. J Cancer Policy. 2024;41:100488. doi:10.1016/j.jcpo.2024.100488 38851632

[47] Lee YC , Chiang TH , Liou JM , Chen HH , Wu MS , Graham DY . Mass eradication of helicobacter pylorito prevent gastric cancer: theoretical and practical considerations. Gut Liver. 2016;10(1):12‐26. doi:10.5009/gnl15091 26696028 PMC4694730

[48] Siddiqi K , Arora M , Gupta PC . Common assumptions in tobacco control that may not hold true for South‐East Asia. Lancet Reg Health Southeast Asia. 2023;8:100088. doi:10.1016/j.lansea.2022.100088 36644450 PMC9831008

